# Ethnological Society, April 23

**Published:** 1845-07

**Authors:** 


### ETHNOLOGICAL SOCIETY, APRIL 23.

Dr. King, read a paper "*On the human mouth*" by Mr. Nasmyth, "Was mankind originally of a low or of an elevated degree of development?" enquires Mr. Nasmyth, and he answers, the development compatible with the due fulfilment of the exactions required from such a being as man must have been perfect. No feature bears so instructively on the solution of the various difficult problems involved in the study of ethnology as the form of the mouth, and the development of the teeth. In the lower animals the mouth is peculiarly and beautifully adapted to their exigencies, but in that of man exists a medium type fitted to every peculiarity of terrestrial existence. No other conformation than that given to him, can at once admit of perfect articulation and mastication of his varied food; moreover it may be regarded as fulfilling a most essential part in his intellectual life, for it is the organ of intellectual expression, and a grand agent in the communion of social life. Deviations in the character of the mouth, Mr. Nasmyth contends, are simply the effects of deviations in the habits of individuals composing races. When these deviations are partial, they are shown in individuals, when general, they amount to a national or tribe characteristic, and when continued from generation to generation, they become hereditary. The natural action of the lower jaw upon the upper is to push out, evert, or expand, the arch of the upper jaw, while on the other hand it is impossible by any habit to bring in or to contract that arch, so as to produce out of the advanced jaw of the negro the vertical jaw of the Caucasian and other well developed races; a vertical is said to be the original development of the infant negro; the advanced mouth of the adult negro is therefore not congenital but factitious. The negro of the southern provinces of the United States, owing to the different circumstances in which he is placed, has not the advanced mouth of his progenitors of Africa, after the 2nd or 3rd generations. Mr. Nasmyth then proceeded to show most ably that the plasticity of the mouth in infancy, was such as to admit of the factitious development pointed out. The ordinary duties required of the mouth in civilized life are a moderate exercise of power for division, tearing, and comminution or grinding, whilst in uncivilized life there exists much more powerful exactions, which have a great controlling influence over the development of the parts. Man in the uncivilized state has but few instruments or tools to assist him in operations of any kind, and his teeth are ready substitutes for those, which, on all occasions from infancy to old age, he most inscrupulously resorts to. He attacks the roughest materials of all kinds with his teeth. He uses them to form and to fashion those materials in all sorts of ways; and thus he converts the dental organ into a prehensile one. He also uses his teeth as instruments for punishing his enemies, seizing his prey, and separating the assimilative portions of his food from those which are not, which with the little assistance he derives from cooking, tend most decidedly to evert both the upper and the under jaw. Mr. Nasmyth explained at length various modifications of the face, arising out of the eversion of the upper jaw so common in uncivilized life, whilst in the civilized, a perfect organization of the mouth was pretty generally accompanied by a well developed brain, a regularity of feature, great energy of character, and corresponding physical power and activity. After the reading of this paper which was as elaborate as it was important, as affecting materially the existing classification of mankind, Dr. Wolff addressed the society on the Asiatic Tribes of his acquaintance, the Turkomans holding a prominent place.—*London Medical Times.*

